# Comparison of Glutathione, Cysteine, and Their Redox Potentials in the Plasma of Critically Ill and Healthy Children

**DOI:** 10.3389/fped.2015.00046

**Published:** 2015-05-26

**Authors:** Jocelyn R. Grunwell, Scott E. Gillespie, Janine M. Ward, Anne M. Fitzpatrick, Lou Ann Brown, Theresa W. Gauthier, Kiran B. Hebbar

**Affiliations:** ^1^Division of Pediatric Critical Care Medicine, Department of Pediatrics, Children’s Healthcare of Atlanta, Emory University School of Medicine, Atlanta, GA, USA; ^2^Division of Infectious Disease, Department of Pediatrics, Emory University School of Medicine, Atlanta, GA, USA; ^3^Division of Neonatal-Perinatal Medicine, Department of Pediatrics, Emory University School of Medicine, Atlanta, GA, USA; ^4^Division of Pulmonology, Allergy & Immunology, Cystic Fibrosis, and Sleep, Department of Pediatrics, Emory University School of Medicine, Atlanta, GA, USA

**Keywords:** oxidative stress, pediatric, critical illness, glutathione, cysteine, redox potential

## Abstract

**Background:**

Oxidative stress is known to play a role in critical illness due to an imbalance in reactive oxygen species and reactive nitrogen species, and the body’s ability to detoxify pro-oxidants using small molecule anti-oxidants and anti-oxidant enzymes.

**Objective:**

To compare the concentrations of plasma redox metabolites and redox potentials for the Cys/CySS and GSH/GSSG thiol/disulfide pairs in critically ill children with healthy control children.

**Methods:**

We performed a prospective clinical observational study of children ages ≤18 years and weight ≥6 kg, who were hospitalized between January 2010 and April 2012 in a 30-bed multidisciplinary medical-surgical pediatric intensive care unit (PICU). We measured the plasma concentrations of Cys, CySS, GSH, and GSSG within the first 24 h of PICU arrival, and we calculated the redox potential for the Cys/CySS (E_h_ Cys/CySS) and GSH/GSSG (E_h_ GSH/GSSG) thiol/disulfide pairs in the plasma of 61 critically ill children and 16 healthy control children.

**Results:**

Critically ill children have less Cys (*p* = 0.009), less CySS (*p* = 0.011), less Total Cys ([Cys] + 2[CySS], *p* = 0.01), more GSSG (*p* < 0.001), and more oxidized E_h_ GSH/GSSG (*p* < 0.001) compared to healthy children.

**Conclusion:**

Our results demonstrate that in the presence of pediatric critical illness, the Total Cys/CySS thiol pool decreases while GSH is likely one component of the cellular redox system that reduces CySS back to Cys, thus maintaining E_h_ Cys/CySS. The Total Cys pool is more abundant than the Total GSH pool in the plasma of children. Further investigation is needed to elucidate the differences in redox potentials in subgroups of critically ill children, and to determine whether differences in redox metabolite concentrations and redox potentials correlate with severity of critical illness and clinical outcomes.

## Introduction

Oxidative stress (OS) causes irreversible damage to DNA, lipids, and proteins (1–3), and is defined as a disruption of redox signaling and control (4–6). The thiols cysteine (Cys) and glutathione (GSH) are common sources of reducing equivalents for neutralizing oxidative stress. Critical Cys residues of many enzymes, receptors, ion channels, transporters, and transcription factors also sense oxidative stress and the oxidative state of these Cys residues influence protein function (4, 5, 7–9). While other small molecule anti-oxidants such as ascorbic acid, uric acid, tocopherols, and carotenoids are important in maintaining redox state of cells, the relative contribution of low molecular weight thiols to total antioxidant capacity is not clear. In addition, the most abundant thiols available for reducing equivalents in serum are albumin and other protein thiols (10, 11).

Cysteine undergoes rapid autoxidation and is toxic to mammalian cells at concentrations exceeding the normal, low micromolar concentrations found in plasma (12). The toxicity of Cys is controlled by incorporating cysteine into the tripeptide GSH (13). While Cys and CySS constitute the predominant low-molecular-weight thiol-disulfide pool in human plasma (14), GSH is more likely to remain reduced in an oxidative environment than Cys. GSH is also maintained in tissues at millimolar concentration with a relatively reduced redox state (15), indicating that the Cys/CySS and GSH/GSSG pool are not in equilibrium (14).

Clinical experience in adults has demonstrated that redox balances of Cys and its disulfide cystine (CySS), and GSH and its glutathione disulfide (GSSG) reflect antioxidant function (16). However, the redox balance of plasma Cys/CySS and GSH/GSSG have not been well characterized in critically ill children. This study compared the concentrations of Cys, CySS, GSH, GSSG, and the redox potentials of the Cys/CySS (E_h_ Cys/CySS) and GSH/GSSG (E_h_ GSH/GSSG) couples in plasma of healthy and critically ill children. Once a difference between redox potentials between healthy control and critically ill children is established, correlation of these redox metabolites with diagnoses, severity of illness scores, and clinical outcomes may lead to the development of new biomarkers of pediatric critical illness.

## Patients, Materials, and Methods

We performed a prospective clinical observational study in the pediatric intensive care unit (PICU) at Children’s Healthcare of Atlanta at Egleston between January 2010 and April 2012. The PICU is a 30-bed multidisciplinary medical-surgical ICU providing quaternary care for patients 0–21 years of age. This study was approved by the Institutional Review Boards of Emory University and Children’s Healthcare of Atlanta. Informed consent was obtained from each patient’s guardian.

### Human subjects

Pediatric intensive care unit (PICU) patients aged 0–18 years weighing ≥6 kg were eligible for enrollment. Blood draws were performed within 24 h of PICU admission. Healthy control children were recruited by advertisement for comparison with PICU patients, and controls were not compensated for their participation in this study. Exclusion criteria for controls included any medication use and a physician diagnosis of chronic disease. Control children were evaluated during an outpatient-only research visit that was rescheduled if upper respiratory infection or other illnesses were present.

Demographic and clinical data such as age, gender, race, primary diagnosis, duration of mechanical ventilation, length of pediatric intensive care unit stay (PICU LOS), length of hospital stay (Hospital LOS), need for advanced technologies such as extracorporeal membranous oxygenation (ECMO), continuous veno-venous hemofiltration (CVVH), and plasma exchange (PE) were noted.

### Determination of severity of illness

Septic shock was defined and classified according to the American College of Critical Care Medicine (ACCM) definitions of cardiovascular support (17). Severity of illness scores were calculated using the Pediatric Risk of Mortality score III (PRISM III) and pediatric logistic organ dysfunction (PELOD) score (18–20).

### Sample collection, processing, and redox state calculations

After obtaining informed consent, plasma samples were immediately placed on ice, and transported to the Emory+Children’s Biomarker Core laboratory. Samples were placed in derivatization solution to stop oxidation and analyses of plasma were performed using high performance liquid chromatography as previously described (21–23). Briefly, blood was collected from controls by peripheral venipuncture or from PICU patients by accessing a central venous line, and 0.25 ml was immediately transferred to a microcentrifuge tube containing 0.25 ml of a preservation solution containing 100 mM serine-borate (pH 8.5) containing (per ml) 0.5 mg sodium heparin, 1 mg bathophenanthroline disulfonate sodium salt (BPDS), and 2 mg iodoacetic acid (21, 22). This procedure has been shown to minimize autoxidation and hemolysis (22). Following centrifugation to remove blood cells, aliquots (125 μl) were transferred to tubes containing 125 μl of 10% (w/v) perchloric acid containing 0.2M boric acid and 10 μM γ-l- glutamyl-l-glutamate as an internal standard. Samples were stored at −80°C prior to further processing to form *N*-dansyl derivatives and analysis by HPLC with fluorescence detection. Metabolites were identified by co-elution with standards, and quantification was obtained by integration relative to the internal standard (22). Total Cys and Total GSH were calculated using the equations: [Cys] + 2[CySS] and [GSH] + 2[GSSG], respectively. The redox states (E_h_) of the thiol/disulfide pools were calculated with the Nernst equation using standard potentials for the GSH/GSSG (−264 mV) and Cys/CySS (−250 mV) pairs at pH 7.4, as previously described (16, 24).

### Statistical methods

All statistical analyses were performed using SAS 9.3 (Cary, NC, USA) and statistical significance was assessed at the 0.05 significance level unless otherwise noted. Since specific differences in metabolite and redox values are difficult to predict, statistical power was evaluated by utilization of an effect size. Samples of 61 cases and 16 controls were obtained giving 80% power to detect an effect size of 0.8 using a two-sample Wilcoxon-test. Prior to the analysis, the assumption of normality for the metabolite and redox values was assessed using the Shapiro-Wilk test for normality and by visual inspection of the histograms. As has been previously reported (16), evaluation of metabolite data demonstrated that none of the metabolites, with the exception of the redox potentials (E_h_), were normally distributed, and thus, values were log transformed and adjusted for differences in age, gender, and race using generalized linear models. Back-transformed least-squares means and 95% confidence intervals are reported. Discrete variables are shown by frequency counts and percentages.

## Results

### Subjects and clinical characteristics

Demographic data are listed in Table 1. Primary diagnoses in the category labeled “Other” included: severe head trauma, post-operative from spinal fusion surgery, cerebral hypertension, tuberculosis, status epilepticus, brain abscess, post-bronchial foreign body, pertussis, traumatic brain injury, acute chest syndrome, parapneumonic effusion, neutropenic fever, and demyelinating central nervous system disease.

**Table 1:** **Demographics, primary diagnosis, and severity of illness measures**.

Characteristic	PICU (*n* = 61)	Controls (*n* = 16)	*p*
Age (year), median (IQR)	11.5 (6.1–16.4)	9.7 (8.4–11.5)	0.697
Gender, *n* (%)			
Male	36 (59)	3 (19)	0.005
Female	25 (41)	13 (81)	
Race, *n* (%)			
African American	38 (62.3)	10 (62.4)	
Caucasian	18 (29.5)	3 (18.8)	
Multiple races	3 (4.9)	0 (0)	0.107
Asian	1 (1.6)	3 (18.8)	
Pacific Islander	1 (1.6)	0 (0)	
Ethnicity, *n* (%)			
Hispanic	4 (6.6)	0 (0)	
Primary diagnosis, *n* (%)			
Septic Shock	24 (39.3)		
Asthma	11 (18.0)		
Shock	8 (13.1)	NA	
Sepsis	4 (6.6)		
Other	14 (23.0)		
Oncology^a^, *n* (%)	9 (12.9)		
Solid tumor	5 (55.6)	NA	
Hematological	4 (44.4)		
Severity of illness measures, mean (SD)			
PRISM III score	11.1 (5.3)	NA	
PELOD score	15.5 (13.7)		
Length of stay (days), median (IQR)			
PICU	6.0 (3.0–9.0)	NA	
Hospital	9.0 (5.0–19.0)		
Mechanically ventilated, *n* (%)	37 (60.7)	NA	
Time on ventilator (days), median (IQR)	6.0 (3.0–8.0)	NA	
Advanced technologies^b^, *n* (%)	10 (16.4)		
ECMO	5 (50)		
CVVH	8 (80)	NA	
PE	3 (30)		
28 days mortality, *n* (%)	3 (4.9)	NA	

*IQR, 25th–75th interquartile range*.

*NA, not applicable*.

*^a^Eight oncology patients had a primary diagnosis of septic shock and one had an “Other” diagnosis of neutropenic fever*.

*^b^ECMO, extracorporeal membrane oxygenation; CVVH, continuous venovenous hemodialysis; PE, plasma exchange. The total percentage of patients receiving advanced technologies is >100%, because some patients received more than one type of advanced technology*.

### Redox status of PICU subjects compared with healthy children

**Figure 1:**
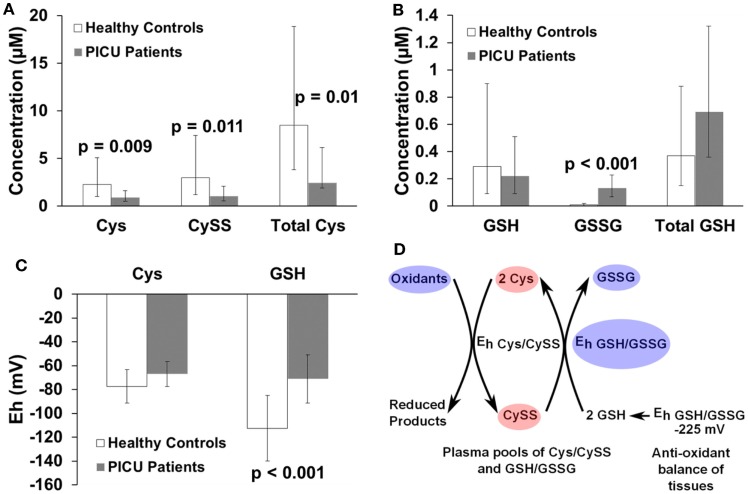
**Bar graphs comparing concentrations of (A) Cys (μM), CySS (μM), and Total Cys (μM); (B) GSH (μM), GSSG (μM), and Total GSH (μM); (C) E_h_ Cys/CySS (mV) and E_h_ GSH/GSSG (mV) of healthy children controls with PICU patients**. Individual bars indicate least-squares mean values and the whiskers represent 95% confidence intervals. Metabolite concentration data were log-transformed, and the back-transformed least-squares means are presented. The E_h_ are normally distributed, and a less negative number reflects a more oxidized redox value. All metabolite and E_h_ values were adjusted for age, gender, and race. *p*-Values are listed above the pairs being compared. **(D)** Represents a model summarizing the redox data in **(A–C)**, adapted from Jones et al. (16). The Total Cys concentration is higher than that of Total GSH. Because Cys is more abundant and more readily oxidized than GSH (as indicated by a less negative redox potential), Cys is preferentially oxidized to CySS in plasma. GSH in the plasma serves as a reducing pool to convert CySS back to Cys, thus maintaining the E_h_ Cys/CySS in critically ill children nearly equal to that of healthy children. The redox balance of Cys is preserved by the supply of GSH from tissues. Red ovals represent a significant decrease in concentration of a metabolite. Blue ovals represent a significant increase in concentration of a metabolite or a more oxidized redox potential (E_h_). Ovals are not representative of the magnitude of change and are not drawn to scale.

Redox data from the PICU subjects and healthy control children are shown in Figure 1. After adjusting for age, gender, and race, children admitted to the PICU had 2.5 times less Cys (2.29 vs. 0.91 μM; *p* = 0.009), 2.8 times less CySS (2.98 vs. 1.05 μM; *p* = 0.011), and 2.5 times less Total Cys (8.52 vs. 3.41 μM; *p* = 0.01) (Figure 1A). Children admitted to the PICU had no difference in GSH concentrations (0.29 vs. 0.22 μM; *p* = 0.573) or Total GSH (0.37 vs. 0.69 μM; *p* = 0.106), but PICU patients had 13 times more GSSG (0.01 vs. 0.13 μM; *p* < 0.001) than healthy control children (Figure 1B). Finally, children admitted to the PICU showed no difference in E_h_ Cys/CySS (−77 vs. −67 mV; *p* = 0.095) compared to healthy children; however, critically ill children had a ~40 mV more oxidized E_h_ GSH/GSSG (−113 vs. −71 mV; *p* < 0.001, Figure 1C) compared to healthy control children.

### Redox status as a function of age, gender, primary diagnosis, severity of illness scores, use of advanced technologies, and clinical outcomes in PICU patients

Children in the PICU with organ dysfunction requiring advanced technologies (*n* = 10) such as ECMO, CVVH, and PE have less Cys [Median (IQR) 0.5 μM (0.3–0.57) vs. 1.02 μM (0.25–2.6); *p* = 0.027], less CySS [0.24 μM (0.1–0.54) vs. 0.79 μM (0.44–3.64); *p* = 0.027], and less Total Cys [1.02 μM (0.64–1.60) vs. 2.85 μM (1.26–11.15); *p* = 0.019] when compared with other children admitted to the PICU (*n* = 51). There was no difference in E_h_ Cys/CySS [Mean (SD) −64 mV (13) vs. −66 mV (7); *p* = 0.911] or any of the GSH redox parameters (*p* > 0.8) measured when comparing the patients receiving advanced technologies with the remaining PICU patients. We compared redox metabolite values, E_h_ Cys/CySS, and E_h_ GSH/GSSG across age and gender and found no significant differences for the PICU patients. There were no strong correlations (ρ ≥ 0.4) significant between primary diagnosis, severity of illness scores, such as PELOD and PRISM III score, or clinical outcomes such as, PICU LOS, Hospital LOS, or days of mechanical ventilation with redox metabolites, E_h_ Cys/CySS, or E_h_ GSH/GSSG.

## Discussion

This study compared the plasma redox metabolite concentrations and redox potentials for the Cys/CySS and GSH/GSSG pairs between critically ill and healthy children. We found that in critically ill children, the total Cys/CySS thiol pool was ~2.5 times less than that in healthy children. Despite the reduction in Total Cys in critically ill children, the E_h_ Cys/CySS in critically ill children was maintained at healthy control levels. However, the E_h_ GSH/GSSG was ~40 mV more oxidized in critically ill vs. healthy children. These findings suggest that while Cys/CySS is the most abundant low-molecular-weight thiol-disulfide pool in plasma (23 times more abundant in plasma of healthy children than the GSH/GSSG pool), GSH is a better reducing agent than Cys as denoted by a more negative redox potential. Therefore, GSH reduces CySS back to Cys in critically ill children, thus maintaining E_h_ Cys/CySS as summarized in Figure 1D, based on a model derived by Jones et al. (16).

This is not the first study to measure cysteine and glutathione concentrations in children. Lyons and colleagues measured Cys metabolism and glutathione synthesis in 10 septic pediatric patients and compared with 10 post-operative controls (25). Results from the Lyons study showed that there was no difference in Cys or CySS levels when methionine intake was controlled; however, a 60% decrease in GSH synthesis rates was noted in septic patients vs. controls (25). Németh and Boda measured a xanthine oxidase activity index and a GSSG/GSH ratio in infants and children with septic shock and showed that these indices were significantly correlated with each other, with severity of illness scores (PRISM), and were increased in patients in a proinflammatory state (26). Our results extend this work by adding the E_h_ Cys/CySS and E_h_ GSH/GSSH to the literature of pediatric acute critical illness. Redox potential, calculated using the Nernst equation, accounts for the stoichiometry of two electron transfers as two GSH are involved in the oxidation to GSSG (6). As an example, the ratio of GSH/GSSG is also used to quantify reducing force for this thiol/disulfide pair; however, a GSH/GSSG ratio of 100 has very different reducing power for a GSH of 10 mM and a GSSG of 100 μM with an Eh GSH/GSSG of −264 mV vs. a GSH of 100 μM and a GSSG of 1 μM with an Eh GSH/GSSG of −204 mV (27, 28). While many assays exist for measuring individual pro-oxidant and anti-oxidant levels, the reason for quantifying the E_h_ Cys/CySS and E_h_ GSH/GSSG in serum is that it provides an overall measure of the pro-oxidant/anti-oxidant balance (6, 28).

Fitzpatrick and colleagues have measured the E_h_ Cys/CySS and E_h_ GSH/GSSH of mild-to-moderate and severe asthmatics (23). In comparison, PICU patients from the current study (mean E_h_ Cys/CySS −65 mV) have a significantly more oxidized Cys/CySS redox potential than mild-to-moderate asthmatics (mean E_h_ Cys/CySS −95 mV) and are similarly oxidized to severe asthmatics in the Fitzpatrick study (mean E_h_ Cys/CySS −55 mV). The subset of PICU patients admitted with a primary diagnosis of asthma (*n* = 11) tended to be the most oxidized subset of patients in the PICU with a mean E_h_ Cys/CySS −51 mV. For the GSH/GSSG redox state, severe asthmatics in the Fitzpatrick study (23) have a mean E_h_ GSH/GSSH of −90 mV similar to asthmatics admitted to the PICU in this study (E_h_ GSH/GSSG −93 mV). Due to the small numbers of asthmatics enrolled in our study, the E_h_ Cys/CySS and E_h_ GSH/GSSG results of patients admitted to the PICU with a primary diagnosis of asthma are not significantly different when compared with PICU patients with other primary diagnoses.

The current prospective study has several limitations. It is a small single-center study comparing critically ill children with diverse diagnoses. Because of the small number of patients with a variety of diagnoses, the study was not adequately powered to detect correlations between redox markers, clinical diagnoses, severity of illness scores, or outcomes such as length of PICU stay or duration of mechanical ventilation. We measured plasma redox markers, but measurements of local tissue redox metabolites, for example redox metabolites in the lung of asthmatics or in those with acute lung injury, may be a more relevant marker of OS in the microenvironment of the lung. We did not compare other biomarkers of oxidative stress or anti-oxidant potential with E_h_ Cys/CySS and E_h_ GSH/GSSG, such as levels of ascorbic acid, 4-hydroxynonenal, malondialdehyde, F2-isoprostanes, or enzymatic activity of common redox enzymes such as superoxide dismutase, glutathione peroxiredoxin, or thioredoxin. This lack of comparison is not unique to our study, and there are several studies comparing plasma biomarkers of oxidative stress in adults have not resulted in correlations among oxidative stress markers or clinical outcomes (29–33). In addition, a recently published study of lipopolysaccharide (LPS) induced sepsis in a porcine model did not show any correlation of many redox markers with each other or with clinical severity of illness (34). Despite these limitations, our data are in agreement with some of the findings of prior studies in septic children (25, 26), and confirm that significant differences exist in the E_h_ GSH/GSSG, rather than E_h_ Cys/CySS, of children admitted to a PICU when compared to healthy control children.

In summary, a decrease in the abundance of Total Cys, an increase in GSSG, and a more oxidized E_h_ GSH/GSSG may serve as potential markers of OS events in the plasma of critically ill children. Understanding changes that occur in redox metabolites, redox potentials, and redox signaling pathways in childhood diseases may lead to novel prognostic markers and therapeutic targets in pediatric critical illness. Future studies will work toward assessing the role of protein thiols, other markers of oxidative stress, and signal transduction pathways to elucidate the mechanism of oxidative stress in critically ill children. In addition to studying the biological mechanisms of how changes in Eh Cys/CySS and Eh GSH/GSSG correlate with other markers of OS and influence redox sensitive redox signaling pathways, we plan on evaluating whether the balance of Cys/CySS and GSH/GSSG can differentiate subpopulations of critically ill children with differing severity of critical illness.

## Conflict of Interest Statement

The authors Jocelyn R. Grunwell, Scott E. Gillespie, Janine M. Ward, Theresa W. Gauthier, Lou Ann Brown, and Kiran B. Hebbar have indicated they have no financial relationships relevant to the article to disclose. Anne M. Fitzpatrick has received consulting fees from the following companies: Boehringer Ingelheim, Genentech Consulting, GlaxoSmithKline Scientific Advisory Board, MedImmune, Inc., Consulting, and Merck Scientific Advisory Board.

## Abbreviations

Cys, cysteine; CySS, cystine; CySSG, cysteine-glutathione disulfide (mixed disulfide); E_h_, redox state calculated from Nernst equation; GSH, glutathione; GSSG, glutathione disulfide.
